# Transcriptome remodeling of mouse hearts during postnatal cardiac maturation and under proteotoxic stress

**DOI:** 10.1007/s11033-026-11535-1

**Published:** 2026-02-07

**Authors:** Mark Bouska, Mingqi Cai, Yue Xing, Erliang Zeng, Xiang Gao, Xuejun Wang

**Affiliations:** 1 Division of Biomedical and Translational Sciences, University of South Dakota Sanford School of Medicine, Vermillion, SD 57069 USA; 2 Department of Computer Science, Loyola University Chicago, Chicago, IL 60153 USA; 3 Division of Biostatistics and Computational Biology, University of Iowa College of Dentistry, Iowa City, IA 52242 USA; 4 Department of Medicine, Stritch School of Medicine, Loyola University Chicago, Maywood, IL 60153 USA

**Keywords:** Proteotoxic stress, Desmin-related cardiomyopathy, SBK2, FOXN4, CRYAB, Cardiac maturation, Aging transcriptomics

## Abstract

**Background:**

Desmin-related cardiomyopathy (DRC) is a proteotoxic disorder driven by mutations in *DES* and related genes such as *CRYAB*^*R120G*^ (R120G), leading to progressive cardiac dysfunction. While late-stage transcriptomic changes in cardiomyopathy and aging are well studied, early molecular events during postnatal maturation and disease onset remain poorly defined.

**Methods and results:**

Through RNA sequencing of mouse ventricular myocardium at multiple time points, we uncovered novel transcriptional changes associated with postnatal cardiac development in non-transgenic mice, as well as early alterations preceding overt pathology in the R120G-based DRC mice. RT-qPCR and western blotting confirmed the kinase SBK2 was downregulated in multiple DRC mouse models, suggesting a conserved role in disease progression. Comparative analysis of our sequencing datasets and an independent RNA-seq dataset, identified a conserved molecular signature involving autophagy and proteasome pathways, notably including the proteasome subunit *Psmd5*. Profiler enrichment analysis uncovered shared transcription factor binding motifs implicating a previously unrecognized transcriptional regulator in disease progression.

**Conclusions:**

These findings identify *Sbk2*, *Psmd5*, *Scml4*, *Snai3*, and *Foxn4* as novel candidates in DRC pathogenesis. In the non-transgenic heart, data implicate several transcriptional networks governing the shift from cardiac maturation to detrimental aging including *AW551984* and a group of zinc finger C2H2 transcription factors (*Zfp41*, *Zfp273*, *Zfp456*, *Zfp469*, and *Zfp820*). These genes have not been studied in the context of cardiac maturation hinting at an unexplored cardiac regulatory network. These findings may provide novel mechanistic insights into the transition from postnatal cardiac maturation to detrimental cardiac aging and proteotoxic stress.

**Supplementary Information:**

The online version contains supplementary material available at 10.1007/s11033-026-11535-1.

## Introduction

Genomic Instability, Epigenetic Alterations, Disabled Macroautophagy, and Loss of Proteostasis are listed as the primary “driver” Hallmarks of Aging [1]. Loss of proteostasis results in the accumulation of damaged and misfolded proteins categorized as “proteotoxic stress.” The *CryAB*^*R120G*^ transgenic (referred to as R120G hereafter) mouse model of desmin-related cardiomyopathy (DRC) recapitulates the human R120G missense mutation in the CRYAB chaperone protein. It is under control of a cardiomyocyte specific promoter and is widely used to model patient proteotoxic stress, general protein aggregation, and loss of proteostasis [2, 3]. The resultant protein aggregation produces DRC which progresses through hypertrophic/restrictive cardiomyopathy, dilated cardiomyopathy, and leads to premature mortality [4, 5].

Because proteotoxic stress is a progressive process we had sought to identify the pathways and genes involved in the disease progression during cardiac maturation. To this end, RNA sequencing of the R120G heart was performed at 1-, 3-, and 6-months which highlighted novel transcription factors, proteins, and pathways yet to be studied in the context of DRC. This longitudinal approach enabled the identification of targets for genetic and pharmaceutical manipulation to improve outcomes from proteotoxic cardiomyopathies.

The RNA sequencing (RNA-seq) of non-transgenic (NTG) mouse hearts also yielded valuable insights into the transition from cardiac maturation to the detrimental effects of cardiac aging. Often the study of aging involves examining organisms at ages in which negative phenotypic effects are already pronounced. However, the point of minimal biological age (ground zero) has been defined by aging epigenetic clocks as occurring between embryonic day E4.5 and E10.5 in mice. This defines the point at which the onset of biological aging occurs [6]. Cardiac development proceeds from this starting point through the embryonic stages and then proceeds into the final stages of cardiac maturation until adulthood. The 3- to 6-month age in mice corresponds to the 20–30 year age range in humans and this is the interval in which more genes change their expression trajectories than any other age (at least in the brain) [7, 8]. These studies suggest that this is the critical time interval during development in which the transition from maturation to detrimental aging occurs. This makes sense from an evolutionary standpoint as the average maternal age in humans across the last 250,000 years of evolution is estimated to be 26.9 years [9]. Thus, mutations that help to maintain tight regulatory transcriptomic control after age 26.9 are less likely to be passed to the next generation. Concurrently, a few studies have established a “turning point” in transcriptome trajectories which clearly demarcates the transition from body maturation to the detrimental effects of aging. Cardiac maturation in particular consists of major structural, contractile, regulatory, and metabolic changes. Although RNA sequencing, as well as single cell RNA-seq (scRNA-seq), has been performed extensively to study cardiac embryonic development (including the late stages of cardiac decline after ~ 18 months) the driving factors and how they interact during the cardiac maturation period remain one of the overarching questions in cardiac research [10]. Additionally, bulk sequencing of the heart is still useful as there are several biases in cardiac scRNA-seq including, but not limited to, the small percentage full transcriptomes per cell, overabundance of mitochondria in cardiomyocytes, and cardiomyocyte size limitations in droplet based systems (reviewed in Gao et al. 2025) [11].

What is occurring at the transcriptional level just before, during, and after cardiac maturation — possibly the most critical period in the aging process — that ultimately leads to the transition from cardiac maturation to the detrimental effects of aging in the heart?

## Results

### Tissue from mice overexpressing R120G-CRYAB has distinct transcriptomes from non-transgenic age-matched cohorts

**Fig. 1:**
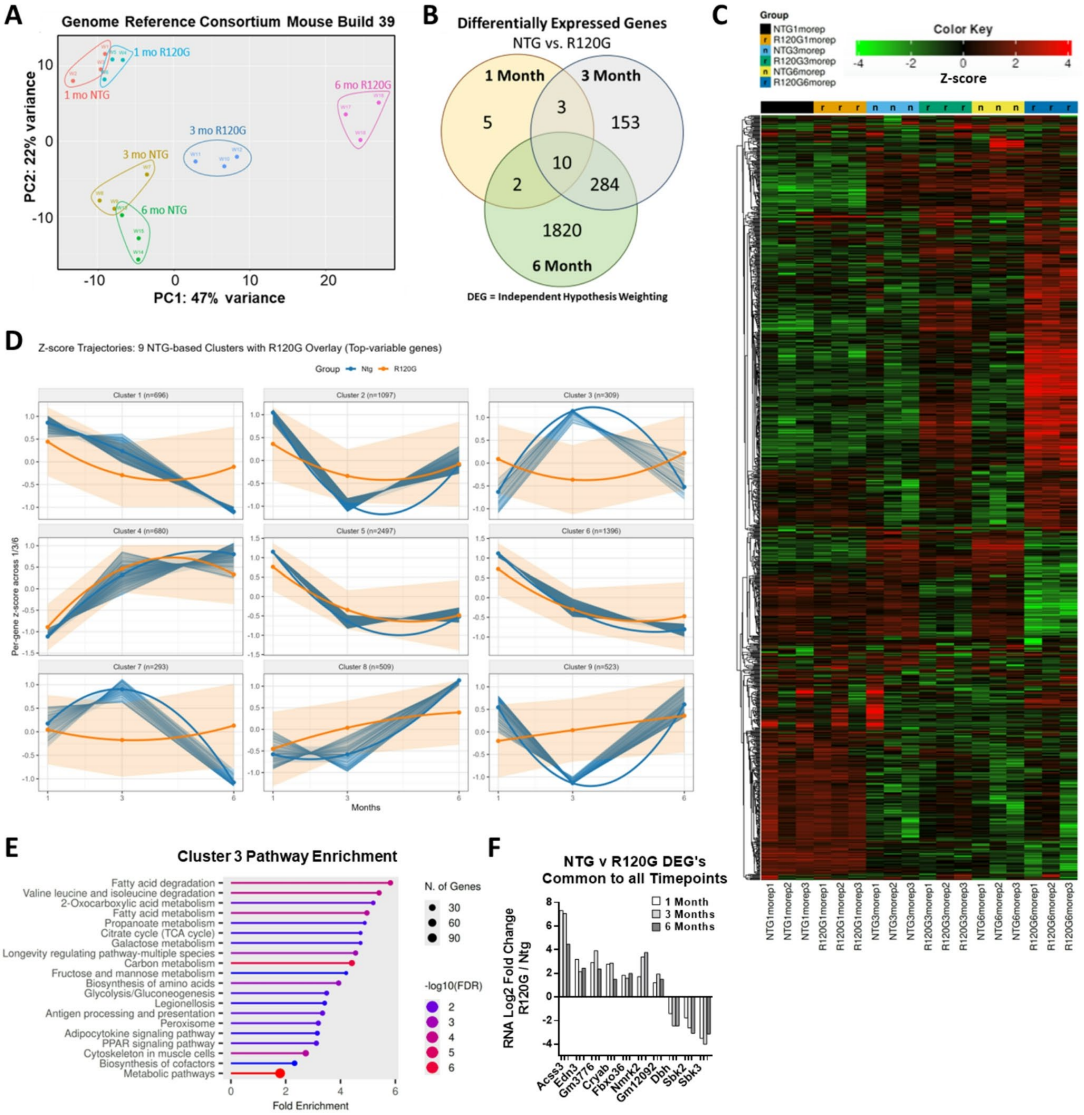
Tissue from mice overexpressing R120G mutant CRYAB has distinct transcriptomes from non-transgenic age-matched cohorts. **(A)** Principal Component Analysis for RNA sequencing of ventricles from 1-, 3-, and 6-month-old non-transgenic (NTG) and R120G (R120G) mice. The first and second principal components for NTG and R120G 1-month old mice are the most similar, and these components diverge further with increasing age (see Figure S2 for calculations). For all RNA sequencing data; *n* = 3 NTG, *n* = 3 R120G male ventricles. **(B)** Differentially expressed genes between NTG and R120G hearts at 3 timepoints. Differential expression was determined using Independent Hypothesis Weighting (IHW) combining Log2 fold change ≥ 1 and adjusted p-values ≤ 0.05. Expression of several genes was validated using RT-qPCR in both males and females (Fig. 4B, others not shown). **(C)** Heatmap of the top 1000 differentially expressed genes using hierarchical clustering and Pearson linkages [12]. **(D)** k-means clustering of the top variable genes from only NTG samples (blue) overlayed with R120G expression trajectories for those same genes (orange). **(E)** Pathway analysis of Cluster 3 displaying Fold Enrichment [12]. **(F)** Relative expression levels of NTG vs. R120G DEG’s common to all 3 timepoints (IHW Log2 fold change ≥ 1 and adjusted p-values ≤ 0.05)

To identify the molecular changes during DRC progression, ventricles from male NTG and R120G littermate mice were collected at 1-, 3-, and 6-months of age. Transcriptional profiling was then performed (GEO repository accession# GSE209839) (Figure S1, Table S1). Principal component analysis (PCA) revealed R120G mouse hearts have distinct transcriptional profiles from NTG age-matched hearts (Fig. 1A), and with age the NTG and R120G profiles increasingly diverge. The numbers of significant differentially expressed genes (DEG’s) also increase dramatically with age (Fig. 1B, Table S2), corresponding to increasing disease severity [2].

To corroborate the PCA grouping data, a heatmap of the top 1000 genes (Hierarchical Clustering) was generated. This highlighted the limited intra-group variability (Fig. 1C, Table S3) [12]. Table S3 also contains Independent Hypothesis Weighting comparisons between each of the various experimental and control groups. Consistency among the biological replicates was evident from large clusters of genes showing similar expression patterns within each group. Heatmapping revealed group-wise similarities between the 3- and 6-month-old R120G groups which also had large distinct blocks of similar gene expression patterns within the 1000 top genes. To further examine the group-wise expression patterns, the top variable genes from NTG samples were z-scored per gene and grouped into 9 clusters using k-means (Fig. 1D, Table S4). The trajectories of identical genes from the R120G hearts were then overlayed. Cluster 3 showed the greatest variance between NTG and R120G, so a pathway enrichment analysis was performed (Fig. 1E).

One unique feature of the data was that only 10 DEG’s occurring between NTG and R120G hearts were common to all 3 timepoints (Fig. 1F). Of these, *Nmrk2*, *Sbk2*, and *Sbk3* all code for kinases that are highly expressed in the heart. Also noteworthy is *Fbxo36*, which codes for an unexplored E3 ubiquitin ligase.

### Dysregulated expression and pathways at 1 month in R120G mice

**Fig. 2:**
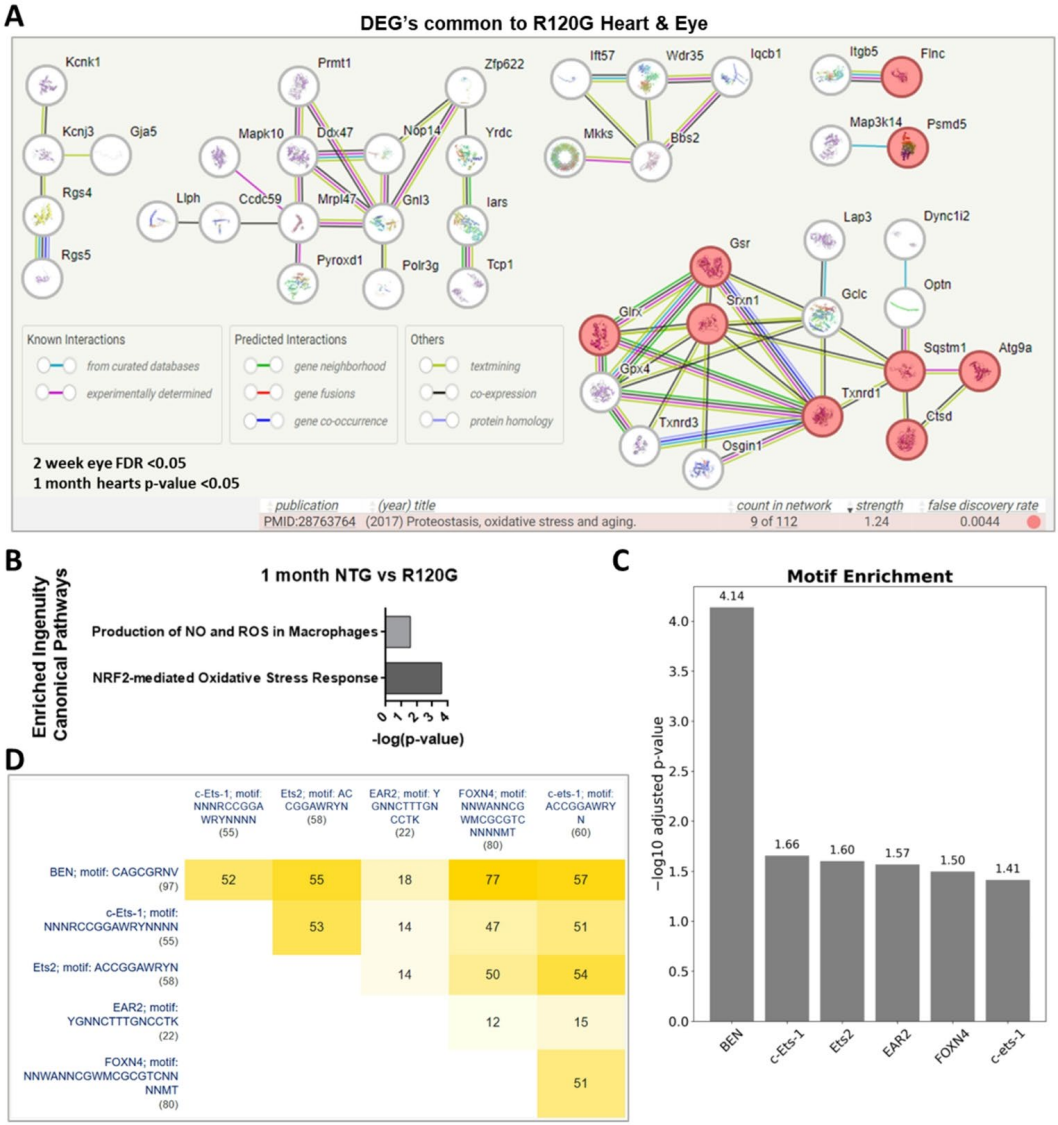
Tissue from mice overexpressing R120G mutant CRYAB has distinct transcriptomes from non-transgenic age-matched cohorts. **(A)** Key nodes among 109 overlapping proteins from our 1-month RNA sequencing and a previous R120G eye RNA sequencing study [12, 13]. Significant eye FDR < 0.05 and heart samples IHW p-value < 0.05 (Table S5). Red colored circles are related to proteostasis, oxidative stress, and aging. For all RNA sequencing data; *n* = 3 NTG, *n* = 3 R120G male ventricles. **(B)** Enriched Ingenuity Pathways from RNA sequencing results for 1-month NTG vs. R120G hearts. Enrichment was determined using QIAGEN Ingenuity Pathway Analysis (-log (p-value)). Right-tailed Fisher’s Exact Test used to calculate p-values [16]. **(C)** Motifs enriched near significant genes shared between R120G heart and eye at 1 month [18]. **(D)** Intersection sizes for transcription factor motifs near shared genes

In cardiac muscle, skeletal muscles, and lens of the eye, CRYAB is a major chaperone protein that is essential to protein quality control including binding misfolded proteins to prevent aberrant protein aggregation. Not surprisingly, the R120G mutation results in protein aggregation. The main mouse models of R120G are used to study cellular responses to increased proteotoxic stress, in particular the ubiquitin-proteasome system (UPS) and autophagy. The eye is the other major organ in which the R120G mutation causes severe aggregation and, in the case of the eye, it results in cataracts. Because both autophagy components and individual proteasome subunits are regulated in response to very specific types of aggregates, we asked if there is a common regulatory network that is activated due to the R120G mutation across tissues. Cross-tissue comparisons tend to generate more comprehensive and robust datasets as well as enhancing the identification of overlooked molecules [13]. We therefore compared our 1-month transcriptomic data with RNA-seq performed on the eyes of 1 month old R120G knock-in mice [14]. We found 110 overlapping proteins with key nodes related to proteotoxic stress, oxidative stress, and aging (Fig. 2A, Table S5) [15].

We next looked at the Ingenuity Canonical Pathways that were enriched [16]. Ingenuity Pathway Analysis looks not only at changes in group-wise expression patterns but also at how genes and gene products interact, thus providing greater mechanistic insights. At the 1-month timepoint, pathways with multiple genes enriched included *NRF2-mediated Oxidative Stress Response* and *Production of Nitric Oxide / Reactive Oxygen Species in Macrophages* (Fig. 2B, Table S6) [17]. Although these pathways are known to be affected in the R120G model, the data reveal several layers of novel connectedness. Importantly, the oxidative stress response via NRF2 appears to drive increased levels of Psmd5. This increase would inhibit proteasome assembly in R120G mice. As a result, proteotoxic stress is exacerbated throughout the lifespan because Psmd5 levels continually rise (Table S1). Inhibiting PSMD5 may represent a viable therapeutic approach to mitigate proteotoxic stress.

To determine common regulatory mechanisms for the 110 genes, g:Profiler enrichment analysis was performed [18]. This returned 6 transcription factor binding motifs that are statistically enriched (Fig. 2C, Table S5). In particular, binding motif for the FOXN4 transcription factor (-log10 adj.*p* = 1.5) was found in 80 of the gene’s regulatory regions, which places FOXN4 as a key node in the response to the R120G mutation. Importantly, multiple transcription factor motifs were common to multiple genes (Fig. 2D), further indicating a common regulatory mechanism behind the response to the R120G mutation.

### Dysregulated expression and pathways in R120G hearts at 3- and 6-months

**Fig. 3:**
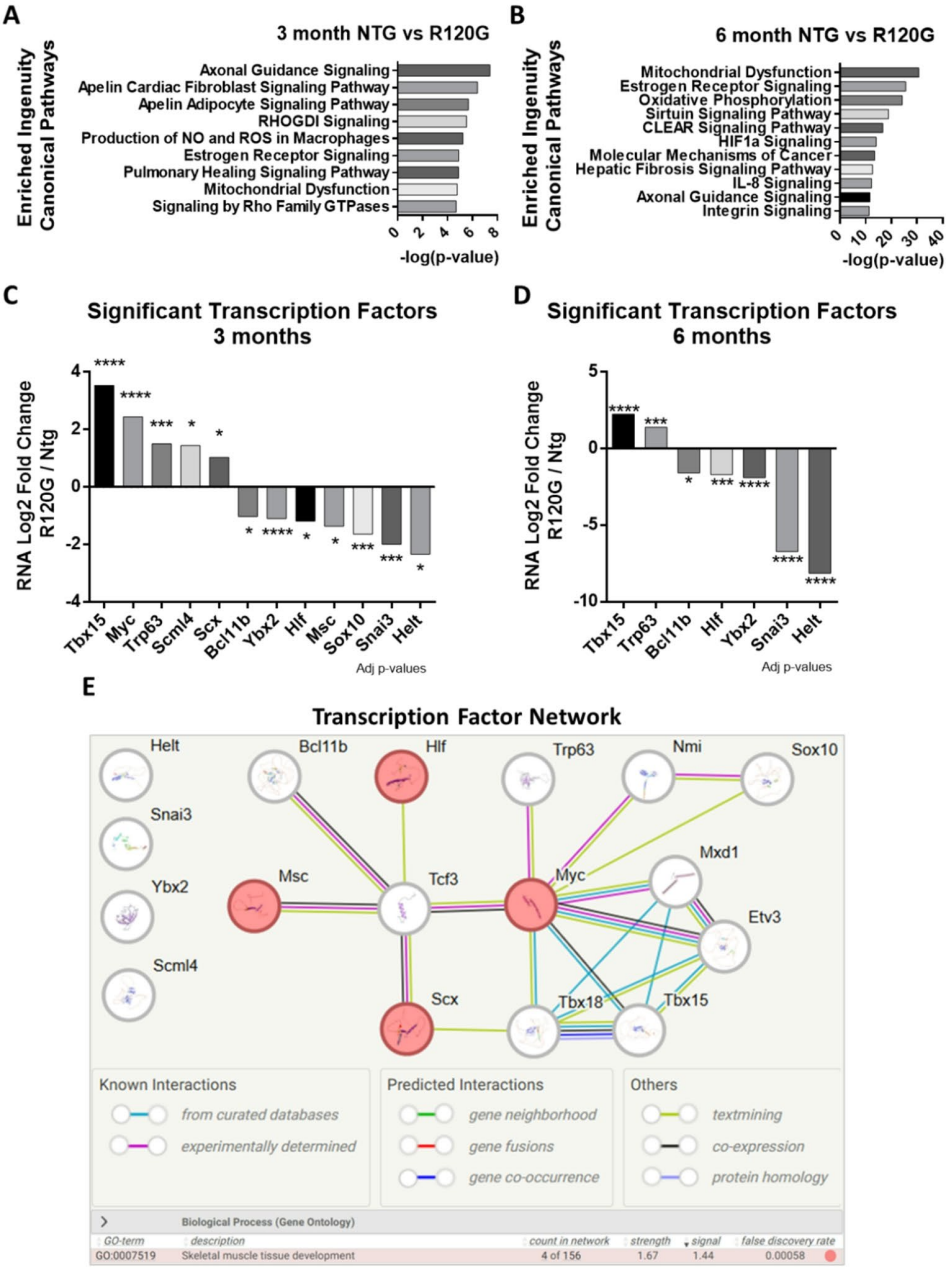
Dysregulated expression and pathways in R120G hearts at 3- and 6-months. **(A-B)** Top Enriched Ingenuity Pathways from RNA sequencing results for 3- and 6-month NTG vs. R120G hearts. Enrichment was determined using QIAGEN Ingenuity Pathway Analysis (-log (p-value)). Right-tailed Fisher’s Exact Test used to calculate p-values [16]. **(C-D)** Significant transcription factors (IHW p-adj. ≤0.05) from RNA sequencing results for 3- and 6-month NTG vs. R120G. *p ≤ 0.05, **p ≤ 0.01, ***p ≤ 0.001****, ns: not significant. **(E)** STRING-db analysis of the 12 significant transcription factors at 3 months was expanded to reveal connections. Expansion of the network revealed 5 proteins (Tcf3, Tbx18, Mxd1, Etv3, and Nmi) created a network based around skeletal muscle development-related networks [15]

At 3 months, there are over 450 DEG’s between NTG and R120G hearts. The top enriched Ingenuity Canonical Pathways revealed several signaling pathways including Apelin Signaling and Estrogen Receptor Signaling (Fig. 3A, Table S6). Apelin levels initially become elevated as a beneficial response to failing hearts but later serve as a biomarker for cardiac dysfunction [19]. Alternatively, Estrogen Receptor Signaling drives mitochondrial biogenesis and a shift to fatty acid oxidation, which in turn promotes the switch from fetal to adult contractile proteins [20]. The reactivation of fetal gene programs occurs in adult hearts with cardiac damage and heart failure. Thus, Apelin Signaling and Estrogen Receptor Signaling reveal that cardiac pathology likely begins between 1- and 3-months in R120G mice.

At 6 months, the top enriched Ingenuity Canonical Pathways between R120G and NTG hearts (Fig. 3B, Table S6) continued to show many of the enriched 3-month pathways but also included pathways related to fibrosis and inflammatory signaling, both of which exacerbate aging processes. These pathways confirm a signature of premature aging in the R120G mouse heart.

We next looked at the significant differentially expressed transcription factors possibly driving the pathways at 3- and 6-months (Fig. 3C-D). *Tbx15* had the highest increase at 3 and 6 months. Transcription factors involved in myogenesis and myo-differentiation including *Snai3*, *Hlf*, and *Msc* were downregulated in R120G hearts at 3-months [21, 22]. Although fewer transcription factors had significant differential expression at 6-months than 3-months, all the 3-months factors were significantly changed again in R120G hearts at 6-months. The transcription factors all continued on their trajectories of continual increase or decrease. Turning off the increasing factors may help alleviate the loss of proteostasis seen in the aging heart following cardiac maturation.

STRING-db analysis revealed that the 12 significant transcription factor DEG’s create a network based around skeletal muscle development-related networks (Fig. 3E). Of the transcription factors in the network, most have known regulatory mechanisms on the heart and have some interactions. However, SCML4’s overall function remains obscure. Even though it has been associated with endothelial cells in vascular disease, it has never been studied directly in the heart. Further examination could reveal a novel player in cardiac function.

### Myocardial SBK2 is downregulated in desmin-related cardiomyopathy

**Fig. 4:**
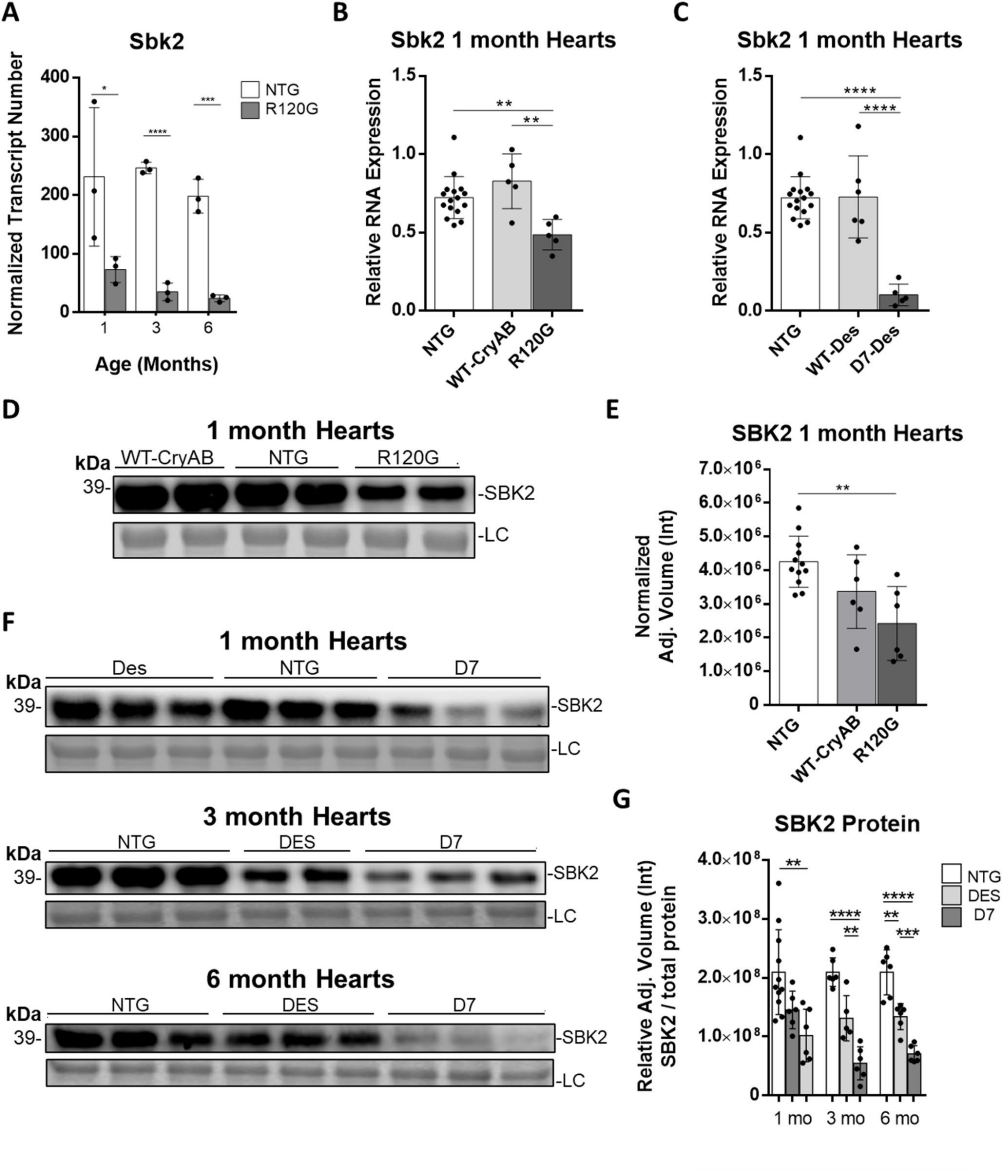
SBK2 is downregulated in desmin-related cardiomyopathy hearts.** (A)** Normalized SBK2 transcript number from RNA sequencing data. Independent Hypothesis Weighting (IHW) combining Log2 fold change ≥ 1 and adjusted p-values ≤ 0.05. **(B)** RT-qPCR for SBK2 from 1-month ventricles from NTG, WT-CRYAB overexpression, and R120G mice (*n* = 16, 5, 5). Unpaired two-tailed t-test *p ≤ 0.05, **p ≤ 0.01, ***p ≤ 0.001, ****p ≤ 0.0001. **(C)** RT-qPCR for SBK2 from 1-month ventricles from male and female NTG, WT-Des overexpression, and D7-Des mice (*n* = 16, 6, 5). Unpaired two-tailed t-test * *p* ≤ 0.05, ** *p* ≤ 0.01, *** *p* ≤ 0.001, **** *p* ≤ 0.0001. **(D)** Representative blot for SBK2 from 1-month ventricles from male and female NTG, WT-CRYAB overexpression, and R120G mice (*n* = 12, 6, 6). **(E)** Quantification of 1 month SBK2 protein levels. One-way Anova Tukey post hoc test * *p* ≤ 0.05, ** p<0.005. Bands normalized to whole gels (Figure S3), LC= loading control. **(F)** Representative blot for SBK2 protein levels from male and female ventricles from NTG, WT-Des overexpression, and D7-Des mice at 1-month (*n* = 11, 6, 6), 3-months (*n* = 5, 5, 6), and 6-months (*n* = 6, 6, 6). **(G)** Quantification of 1-, 3-, and 6-month Western blots from Figure F. One-way Anova Tukey post hoc test * *p* ≤ 0.05, ** *p* ≤ 0.01, *** *p* ≤ 0.001****. Bands normalized to whole gels (Figure S3), LC= loading control

There were only 10 DEG’s between NTG and R120G hearts found at all 3 timepoints (Fig. 1B) which indicated that these are likely to have more direct impact on, or are impacted by, the R120G mutant protein. In particular, *Sbk2* had a significant decrease in mRNA expression in the hearts of R120G mice (Fig. 4A). To determine if the decreased expression of *Sbk2* was due to the R120G mutant and not simply overexpression of CRYAB, RT-qPCR was also carried out on a mouse model that has cardiac specific overexpression of wild-type CRYAB (Fig. 4B). Western blotting and RT-qPCR revealed that the lower *Sbk2* mRNA levels in R120G males and females were due to effects derived from the R120G mutation.

The next question addressed was if the suppression of *Sbk2* was specific to R120G, or could this be related to proteotoxic stress and DRC more generally? Indeed, *Sbk2* mRNA levels were significantly reduced in hearts of the D7-Desmin mutant mouse model of DRC (Fig. 4C). The D7 mouse has cardiomyocyte-restricted overexpression of a Desmin protein containing a 7-amino-acid (R172-E178) deletion and results in protein aggregation and cardiac dysfunction [23]. Significantly lower SBK2 expression in both mouse models was verified at the protein level using western blotting (Fig. 4D-G, S3). This data is the first to reveal that SBK2 is significantly reduced in the hearts of multiple models of DRC.

### Transcriptional networks indicate significant shutdown of structural pathways in NTG heart maturation between 1- to 3-months

**Fig. 5:**
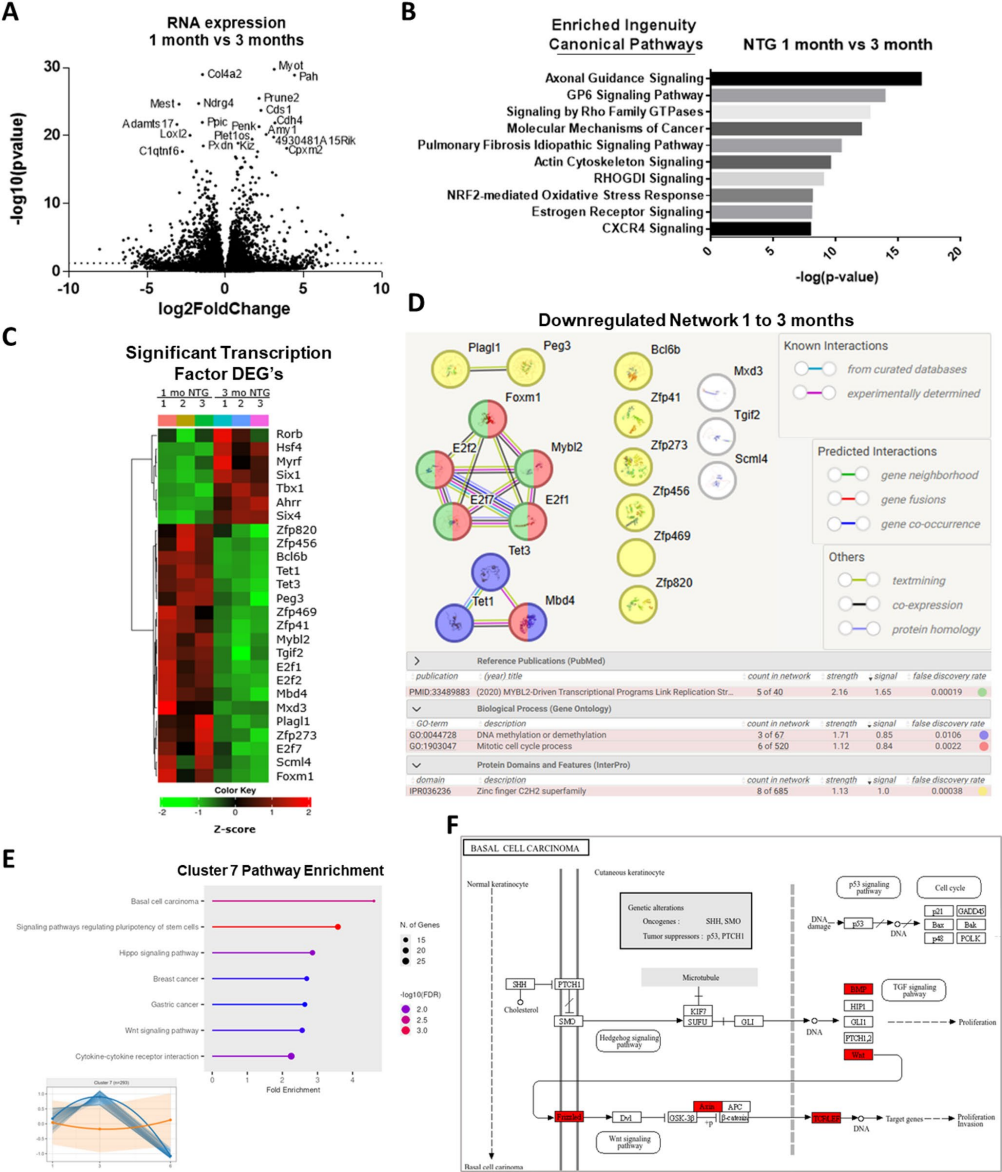
Transcriptional changes in NTG heart from 1 to 3 months. **(A)** From 1- to 3-months there were over 600 significantly DEG’s. IHW combining Log2 fold change ≥ 1 and adjusted p-values ≤ 0.05. (Col4a1, *Adamts12*, *Gm20716*, and *Gm20431* are outside the axis limits). **(B)** Significant Ingenuity Canonical Pathways changing from 1- to 3-months. Enrichment was determined using QIAGEN Ingenuity Pathway Analysis (-log (p-value)). Right-tailed Fisher’s Exact Test used to calculate p-values [16]. **(C)** Heatmap of significant transcription factor DEG’s (IHW p-adj. ≤0.05) between 1- and 3-month NTG hearts grouped using hierarchical clustering [12]. **(D)** STRING-db direct and indirect network of significant downregulated RNA from 1- to 3- months [15]. **(E)** Pathway enrichment for cluster 7 (inset lower left) [12]. **(F)** Kyoto Encyclopedia of Genes and Genomes (KEGG) analysis for top Pathway (Basal Cell Carcinoma) from cluster 7 [12]

We next examined the transcriptional changes due to normal cardiac maturation in NTG hearts. There were over 600 significant DEG changes from 1-month to 3-months (IHW Log2 fold change ≥ 1 and adjusted p-values ≤ 0.05). The most highly altered individual DEG’s include the downregulation of *Loxl2* (Fig. 5A). LOXL2 promotes collagen and elastin crosslinking, is the only one of the top 20 DEGs near one of the 353 CpG sites in the Horvath aging clock, and is a key heart aging molecule [24, 25]. Furthermore, collagens *Col4a1*, *Col4a2*, and *Adamts17* metalloprotease RNA are also highly downregulated. Ingenuity Pathway Analysis showed the top enriched pathways mainly involve actin cytoskeleton and ECM remodeling (Fig. 5B, Table S6). Remodeling was further highlighted by the Rho Family GTPases category (which are master modulators of the actin cytoskeleton), and GP6 signaling category (which involves a receptor for binding and regulating collagen) [26].

Collectively, these results demonstrate that cardiac structural maturation is apparent, but what are the driving factors that are finalizing the maturation process and then leading the heart down a path of detrimental aging? To explore this question, we looked at the transcription factors. The significant differentially expressed transcription factors (Fig. 5C) reveal a molecular picture of key regulatory changes in the heart occurring between 1- and 3-months [12]. The upregulated transcription factor mRNA includes *Tbx1*, *Myrf*, *Six1*, and *Six4.* These are key development and muscle cell differentiation regulators [27, 28]. However, the downregulated transcription factors (Fig. 5C-D) have several important characteristics. First *Tet1*, *Tet3*, (DNA methyltransferases) and *Mbd4* (a glycosylase that leads to demethylation) are decreased. This suggests selective suppression of DNA methylation occurs during the 1- to 3-month period [29, 30]. Second, MYBL2 transcriptional networks are downregulated, indicating reduced mitotic activity and that cardiomyocyte proliferation programs are affected [31, 32]. Third, is that a subset of zinc finger C2H2 transcription factors (*Zfp41*, *Zfp273*, *Zfp456*, and *Zfp820*) were being downregulated. Of these several have not been studied in the heart, hinting at an unexplored cardiac regulatory network.

To determine what pathways were being shut down from 1 to 3 months, but were reactivated later, a pathway analysis of trajectory Cluster 7 (from Fig. 1D) was generated (Fig. 5E). Basal cell carcinoma was the top pathway, which is regulated by the BMP and WNT signaling (Fig. 5F). BMP and WNT signaling are used to derive epicardial cells from human pluripotent stem cells [33].

Collectively, the data indicates that strong regulation of DNA methylation, WNT signaling, and ECM structural changes are occurring in the mouse heart between 1- and 3-months and point to this as the critical time interval for a transition from maturation to the detrimental effects of aging in the mouse heart.

### Genes with significant expression changes from 1- to 3-months and 3- to 6-months in NTG hearts

**Fig. 6:**
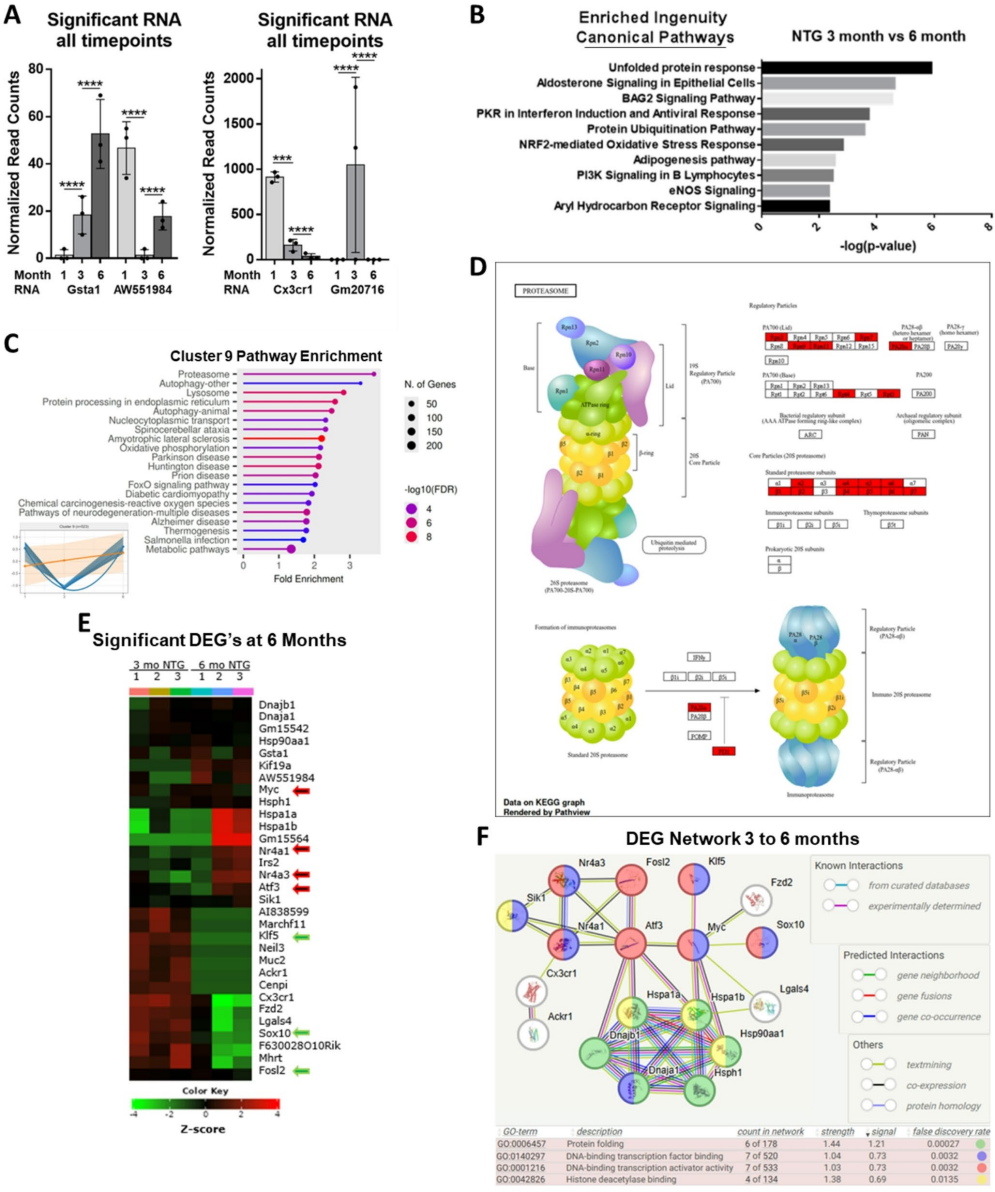
Transcriptional changes in NTG heart from 3- to 6-months. **(A)** Normalized read counts from the only four genes having significant differential expression between all 3 timepoints. One-way Anova Tukey post hoc test *p ≤ 0.05, **p ≤ 0.01, ***p ≤ 0.001****. **(B)** Significant Ingenuity Canonical Pathways changing from 3- to 6-months. Enrichment was determined using QIAGEN Ingenuity Pathway Analysis (-log (p-value)). Right-tailed Fisher’s Exact Test used to calculate p-values [14]. **(C)** Pathway analysis of Cluster 9 (from Fig. 1D) displaying Fold enrichment [12]. **(D)** KEGG analysis of top pathway (Proteasome) from Cluster 9 [12]. **(E)** Heatmap of altered RNA expression from 3- to 6-months. (IHW p-adj. ≤0.05) Transcription factors indicated with red and green arrows; grouped using hierarchical clustering [12]. **(F)** Network of significant DEG’s from 3- to 6-months [15]. DEG’s that do not fall into the network were excluded

The most significant DEG’s occurring from 1- to 3-months that are also significant DEG’s from 3- to 6-months were *Cx3cr1*, *AW554984*, *Gsta1*, and *Gm20716* (Fig. 6A). Of the 4 genes, the 2 genes with extreme turning points include *GM20716*, an autophagy related lncRNA which has extremely high expression only at the 3-month stage, and *AW551984*. Interestingly, we reveal *AW551984* has a change in transcriptome trajectory at the 3-month time period, to the point where it is negligibly expressed at 3 months but then increases by 6 months. The Human protein atlas shows elevated levels of VWA5A (the human homolog of AW551984) from cardiac tissue over age 40, particularly in epicardial cells. Epicardial cells are major players in cardiac repair, as well as in animal models for heart regeneration. Only one study has looked at AW551984’s role in the heart by using stem cells, but it was able to put Wnt signaling upstream of AW551984 and put Nkx2.5 (a critical heart development transcription factor) downstream [34]. BMP and WNT signaling are used to derive epicardial cells from human pluripotent stem cells and are upregulated in cluster 7 (Figs. 5E-D) [33]. STRING-db also indicates AW551984 may play a role in histone variant regulation but this has never been investigated. Cumulatively, the data points to AW551984 as having an underexplored role in the transition from cardiac maturation to the detrimental effects of aging.

### Protein quality control and transcription factors change in heart between 3- to 6-months

The top enriched Ingenuity Canonical Pathways changing from 3- to 6-months confirm that protein homeostasis becomes the defining feature of the aging heart (Fig. 6B). The unfolded protein response (UPR), Bag2 Signaling Pathway, and Protein Ubiquitin Pathways are dominant pathways. The Nrf2-mediated Oxidative Stress Response also becomes enriched, which is noteworthy because this pathway was the most significantly increased pathway at 1-month in R120G hearts (Fig. 2B). Pathway analysis of Cluster 9 showed that both proteasome and autophagy are the main pathways that decline at 3 months and then return to increased expression at 6 months (Fig. 6C-D) to levels seen in the R120G mouse.

We next analyzed only the significant DEG’s from 3- to 6-months (Fig. 6E). Following protein folding related categories, the Gene Ontology Molecular Function category “transcription factor activity” was significantly overrepresented (*p* = 0.0001) (Fig. 6E). The transcription factors *Sox10*, *Fosl2*, and *Klf5* decrease while *Atf3*, *Myc*, *Nr4a1*, *Nr4a3* increase. *Sox10* expression is reduced by half and *Klf5* expression drops to zero. Interestingly, analysis of chromatin immunoprecipitation assays using TFLink revealed that KLF5 targets regulatory sequences in DNA for all of the other significant transcription factors both up and down regulated (except *Sox10*), while FOSL2 targets all of the upregulated transcription factors except *Myc* [35]. KLF5 and FOSL2 are both critical components for Kras signaling, and their decline further corroborates a decline in cell growth and division [36].

The significant DEG’s from 3- to 6-months form a network that has significantly more interactions than expected (PPI enrichment p-value: 3.33e-16) and include an abundance of heat shock proteins related to aging (Fig. 6F) [15]. The heat shock proteins regulate ubiquitination and/or play a role in determining the routing of ubiquitinated proteins to the proteasomal, aggresomal, or autophagic pathways. Additionally, they interact with the differentially expressed transcription factors at this timepoint.

## Discussion

Transcriptomics data from the aging R120G heart has revealed several new molecules that likely play a role in DRC. In particular, the *Sbk2*, *Sbk3*, and *Nmrk2* kinases are dysregulated at all timepoints in R120G hearts and may be important players in phosphorylation signaling pathways that affect DRC progression. While *Nmrk2* has been shown to be elevated in heart disease, *Sbk2* and *Sbk3* play a role in cardiomyocyte differentiation / development that is only beginning to be uncovered [37, 38]. Herein we showed that SBK2 is downregulated in multiple models of DRC. This finding prompted our group to investigate SBK2’s kinase activity and mechanism of action in the heart, which we subsequently validated in a concurrent cardiac specific SBK2 overexpression and echocardiography study [39].

At 1-month in R120G hearts (and at 6-months during normal aging) the Nrf2 pathway is upregulated. Critically, NRF2 upregulates PSMD5 which in turn inhibits the assembly of the 26 S proteasome [40]. Drugs activating PSMD5 are being heavily pursued for treating cancer, inversely PSMD5 inhibitors could be promising in the context of delaying the effects of DRC or aging.

The significant transcription factors at 3-months in R120G hearts reveal that dilated cardiomyopathy has begun. *Tbx15* is the most highly elevated transcription factor at this time. TBX15 regulates a gene network that induces dilated cardiomyopathy [41]. However, we also found that the *Snai3* transcription factor was suppressed at both 3 and 6 months. This observation is consistent with previous reports showing that mutations in Snai3 are associated with genetic cardiac hypertrophy [22, 42]. Further investigation of *Snai3* should therefore provide novel insight into how dilated cardiomyopathy and hypertrophy are transcriptionally regulated.

Although we cannot rule out the possibility that our findings are due to indirect secondary effects from secondary cell types or tissue–tissue interactions, our data are consistent with effects from the cardiomyocyte specific expression of CRYAB-R120G [2, 5, 23]. In myocardium, cardiomyocytes take approximately 80% of the tissue volume; so, the observed transcriptomic changes more likely reflect alterations of and changes resulting from changes occurred in the cardiomyocytes compartment. Indeed, no cells are in isolation in the body. It is well characterized that the R120G mice used here show no or very minimal abnormality (low levels of aberrant protein aggregates in cardiomyocytes) at 1 month of age. Hence, the inclusion of the 1- 3- and 6-month time points help to decipher the early changes that are unlikely from cardiac dysfunction. These data suggest that the mechanistic basis of the other factors identified in this study warrants further investigation.

The longitudinal transcriptomics data from the NTG heart has revealed novel transcriptional networks that are likely drivers of the transition from heart maturation to the detrimental effects of aging. Particularly, we have highlighted a cluster of downregulated zfp transcription factors (*Zfp41*, *Zfp273*, *Zfp456*, and *Zfp820*) that contain KRAB domains, which are strong repressors of transcription [43]. This brings up an intriguing possibility that at least part of the release of repression networks at the critical 3 month timepoint could be due to these factors. Loss of repression is a constant problem during late stage aging. Additionally, Zfp469 has been shown to be a regulator of collagen in the eye [44]. This could also be an important finding as cardiac fibrosis is a feature of aging even in the absence of cardiac injury. Because these transcription factors have not been examined in the aging heart, further investigation may provide novel mechanistic insights into the transition from cardiac maturation to the detrimental effects of aging in the heart.

## Methods

### Animals

FVB/NJ (FVB or NTG) (Jackson Laboratories Strain #001800) females were crossed with heterozygous males containing WT-CRYAB (line 11), R120G (line 134), WT-Des (line 520), or D7-Des (line 641) overexpression and resultant pups were used in this study [2, 23].

### RNA-sequencing

Total RNA was isolated from ventricular myocardium of R120G mice and NTG littermates using the RNeasy Fibrous Tissue Mini Kit (QIAGEN# 74704). Nanodrop assay (Nanodrop 2000, ThermoFisher Scientific) was used to detect the concentration of RNA. RNA-seq libraries were prepared with TruSeq stranded mRNA RNA library prep kit and sequenced using Novase6000 S4 150PE. RNA quality check, library preparation, and sequencing were performed by Psomagen (Psomagen Inc., Rockville, MD, USA).

RNA sequencing data were deposited to the GEO repository under accession number GSE209839. Data were processed using the following workflow: Quality control (Fastqc), Trim adapters and remove low quality sequences (Trimmomatic), Build index (GRCm39.106) (Hisat2), Alignment (Hisat2, Samtools), Raw count table (Stringtie), Normalized count tables and pairwise DE comparisons (DESeq2) [45–49]. Logs, and scripts can be found in supplemental materials (Figure S2).

For gene expression trajectory clustering, normalized RNA-seq counts were log-transformed (log1p) and averaged across replicates for each gene at 1-, 3-, and 6- months. The top variable genes from NTG samples were z-scored per gene and grouped into 9 clusters using k-means (Euclidean distance, k = 9, nstart = 100). R120G trajectories for the same genes were then overlaid. z-score trajectories are shown as spaghetti plots for NTG, with cluster-level median z-scores for NTG and R120G overlaid as smoothed curves (cubic spline) and interquartile range ribbons. Plots were generated in R (v4.x) using tidyverse and ggplot2. ShinyGO, Pathview, and KEGG were utilized to analyze clusters and generate cluster analysis figures.

Pathway analysis was performed using QIAGEN Ingenuity Pathway Analysis (https://digitalinsights.qiagen.com/products-overview/discovery-insights-portfolio/analysis-and-visualization/qiagen-ipa/). Briefly, pathway significance was determined by the number of differentially expressed genes between NTG and R120G in the pathway (at the tested time points) divided by total number of genes in the pathway; combined with right-tailed Fisher’s Exact Test to calculate p-values. The algorithms developed for use in QIAGEN IPA can be found in Kramer et al. 2014 [16].

### RT-qPCR

RNA was extracted using tri-reagent (Invitrogen AM9738), chloroform, and isopropanol, and washed in RNase free ethanol. DNase treatment (Promega M6101) was performed following manufacturer protocol. RNA was quantified using Thermo Scientific Nanodrop 2000 Spectrophotometer. cDNA was synthesized using the High-Capacity cDNA Reverse Transcription Kit (Applied Biosystems #4308228) on the Bio-Rad T100 thermal cycler. qPCR was performed using PowerUp SYBR Green Master Mix (Thermo Fisher# A25742) on Applied Biosystems QuantStudio 5 following manufacturer protocols. Primers for SBK2 (ggaggcctaacggtagagga, ggcactcagagccatcatgt) and GAPDH (tcaacagcaactcccactcttcca, accctgttgctgtagccgtattca) were used. SBK2 relative expression was calculated using the ΔΔCT method.

### Western blotting

Tissues were bead blended (Next Advance Bullet Blender Storm Pro) on high for 5 min in 1X lysis buffer (0.5 mL 3 M Tris HCl pH 6.8, 4.5 mL 10% SDS, 3.0 mL Glycerol, and 28.5 mL H2O) containing Protease inhibitor cocktail (Boster Bio AR1182-1). Samples were boiled for 5 min then centrifuged at 14,000 rpm for 20 min. Proteins were quantified using BCA. Gels were run at 110 V and then wet-transferred for at 250mAmps for 140 min at 4 degrees. Membranes were blocked in 5% BSA in 1X TBST for 1 h, then incubated in primary antibodies, α-SBK2 1:500 (Atlas Antibodies HPA030631), overnight. Membranes were then washed 3 times in TBS, incubated in chemiluminescent substrate, and imaged on Bio-Rad Chemidoc MP. Membranes were normalized to whole protein and relative abundance was calculated using Bio-Rad Image Lab Version 6.1 Software.

## Supplementary Information

Below is the link to the electronic supplementary material.

Supplementary Material 1

## Data Availability

RNA sequencing raw data deposited in: NCBI’s Gene Expression Omnibus (GEO) under repository accession# GSE209839.

## Abbreviations

R120G: CRYAB^R120G^
DRC: Desmin-related cardiomyopathy
IHW: Independent hypothesis weighting
KEGG: Kyoto Encyclopedia of Genes and Genomes
NTG: Non-transgenic
RNA-seq: RNA sequencing
scRNA-seq: Single cell RNA-seq
UPS: Ubiquitin-proteasome system
UPR: Unfolded protein response
