# Supplementary material for: Transcriptome remodeling of mouse hearts during postnatal cardiac maturation and under proteotoxic stress
Source: Mol Biol Rep. 2026 Feb 7;53(1):369. doi: 10.1007/s11033-026-11535-1 (PMC12882862; doi:10.1007/s11033-026-11535-1)
Supplement: Supplementary file 1 — Supplementary Material 1 [file 11033_2026_11535_MOESM1_ESM.zip › SBK2 RNA seq Supplementals/Figure S1. RNA sequencing Psomagen RNA Quality Control.docx]

**Figure S1. RNA sequencing Psomagen RNA Quality Control**

**B.**


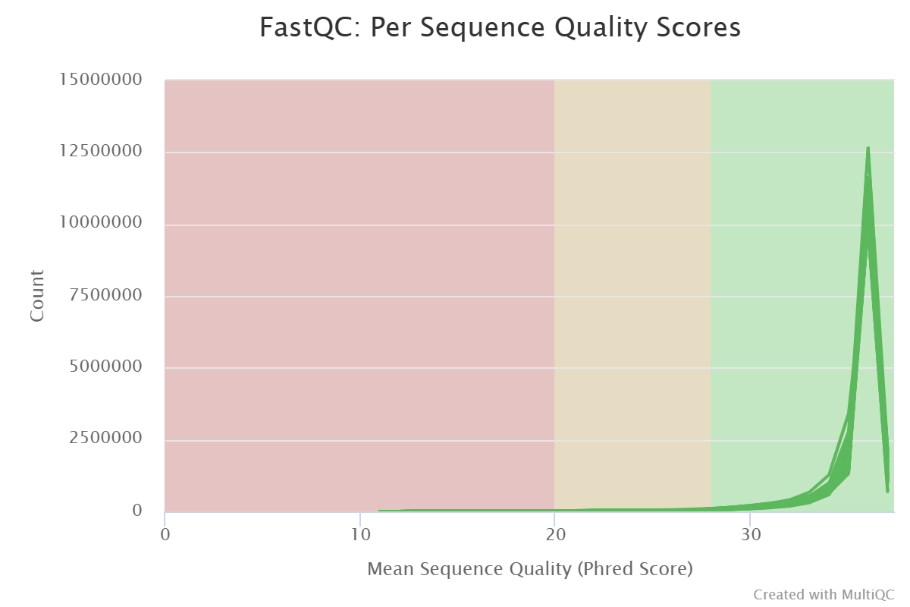


**C.**


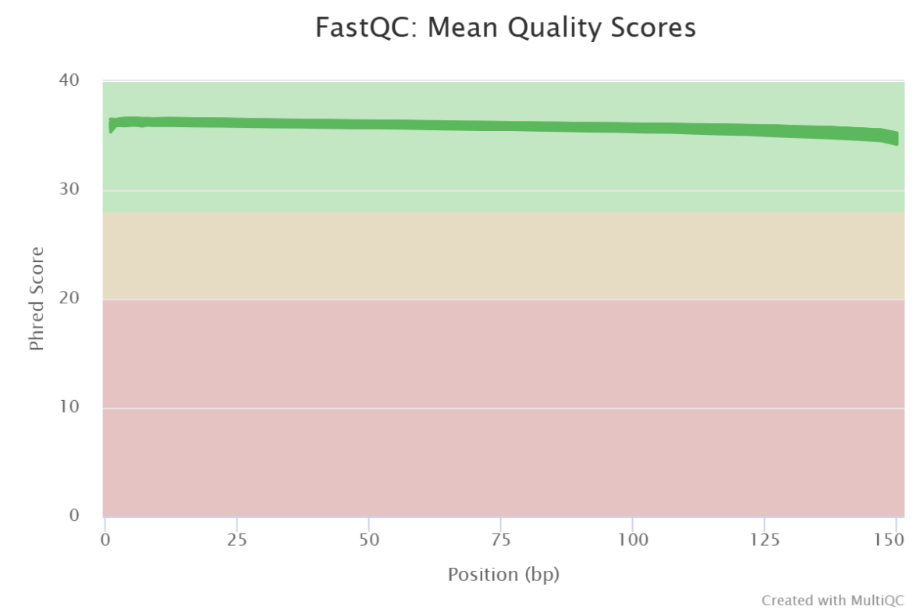


**D.**


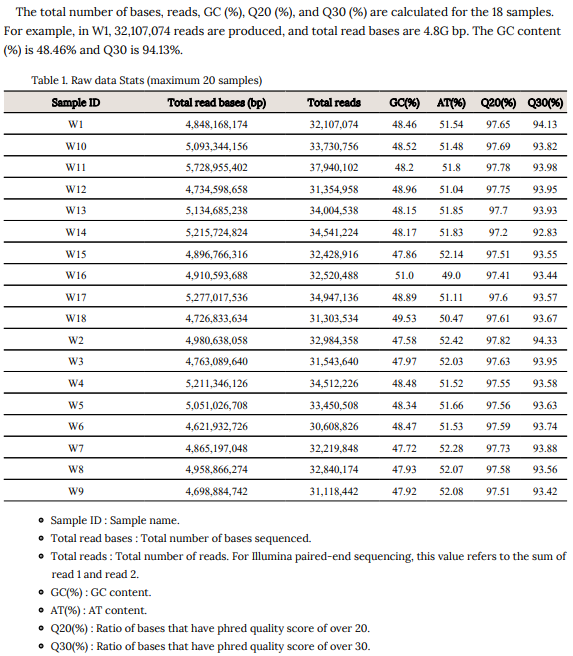

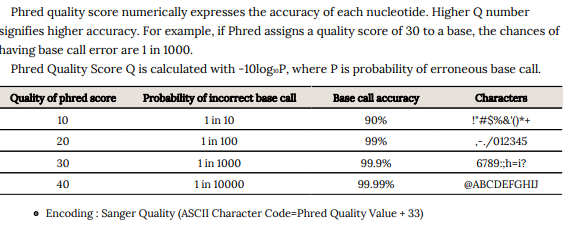


**Figure S1. RNA sequencing Psomagen RNA Quality Control. (A)** Psomagen RNA Quality Control for RNA seq. RNA Integrity numbers (RIN) shown. **(B)** Per Sequence Quality Scores. The number of reads with average quality scores. Shows if a subset of reads has poor quality. **(C)** Sequence Quality Histograms. The mean quality value across each base position in the read. **(D)** Raw Data Statistics.
