# Supplementary material for: Transcriptome remodeling of mouse hearts during postnatal cardiac maturation and under proteotoxic stress
Source: Mol Biol Rep. 2026 Feb 7;53(1):369. doi: 10.1007/s11033-026-11535-1 (PMC12882862; doi:10.1007/s11033-026-11535-1)
Supplement: Supplementary file 1 — Supplementary Material 1 [file 11033_2026_11535_MOESM1_ESM.zip › SBK2 RNA seq Supplementals/Figure S2. Logs and Scripts Process RNA-seq data.docx]

**Figure S2. Logs and Scripts Process RNA-seq data**

# 05/12/2022

# Process FVB/N mice RNA-seq data

### This file processes the RNA-seq sequence data

## Get data

Download the data and save at /N/project/IUMP/dongq10152020/FVBmice/rawfastq.

cp -r /N/project/IUMP/dongq10152020/FVB_mice/raw_fastq /N/project/IUMP/dongq10152020/FVB_mice/fastq cd /N/project/IUMP/dongq10152020/FVB_mice/fastq gunzip * cd /N/project/IUMP/dongq10152020/FVB_mice/raw_fastq ls *_1.fastq.gz |sed 's/_1.fastq.gz//g' > /N/project/IUMP/dongq10152020/FVB_mice/file.list

## Trim adaptor and qc

Merged all adapters to ~/tools/Trimmomatic-0.39/adapters/adapters.fa, and removed duplicates.

cp ~/tools/Trimmomatic-0.39/adapters/adapters.fa .

#### trim.sh

#!/bin/bash #PBS -l nodes=1:ppn=16,walltime=6:00:00,vmem=56gb #PBS -m abe #PBS -M yue.july.xing@gmail.com #PBS -N trim #PBS -o trim.o #PBS -e trim.e cd /N/project/IUMP/dongq10152020/FVB_mice mkdir fq_trim cat file.list | while read name do java -jar ~/tools/Trimmomatic-0.39/trimmomatic-0.39.jar PE \ -threads 10 -trimlog fq_trim/${name}.log \ fastq/${name}_1.fastq fastq/${name}_2.fastq \ -baseout fq_trim/${name}.fq.gz \ ILLUMINACLIP:adapters.fa:2:30:10:2:keepBothReads SLIDINGWINDOW:4:30 MINLEN:30 done

#### fastqc.sh

#!/bin/bash #PBS -l nodes=1:ppn=16,walltime=6:00:00,vmem=56gb #PBS -m abe #PBS -M yue.july.xing@gmail.com #PBS -N fastqc #PBS -o fastqc.o #PBS -e fastqc.e cd /N/project/IUMP/dongq10152020/FVB_mice mkdir fastqc fastqc -t 10 -o fastqc fastq/*

#### fastqc_trim.sh

#!/bin/bash #PBS -l nodes=1:ppn=16,walltime=6:00:00,vmem=56gb #PBS -m abe #PBS -M yue.july.xing@gmail.com #PBS -N fastqc #PBS -o fastqc.o #PBS -e fastqc.e cd /N/project/IUMP/dongq10152020/FVB_mice mkdir fq_trim_qc fastqc -t 10 -o fq_trim_qc fq_trim/*

source activate multiqc multiqc fastqc

Rename outputs.

multiqc fq_trim_qc

Rename outputs.

## Build index

ENSEMBL_RELEASE=106 ENSEMBL_GRCm39_BASE=http://ftp.ensembl.org/pub/release-${ENSEMBL_RELEASE}/fasta/mus_musculus/dna/ ENSEMBL_GRCm39_GTF_BASE=http://ftp.ensembl.org/pub/release-${ENSEMBL_RELEASE}/gtf/mus_musculus/ GTF_FILE=Mus_musculus.GRCm39.106.gtf F=Mus_musculus.GRCm39.dna.primary_assembly.fa wget ${ENSEMBL_GRCm39_BASE}/$F.gz gunzip $F.gz mv $F genome.fa wget ${ENSEMBL_GRCm39_GTF_BASE}/${GTF_FILE}.gz gunzip ${GTF_FILE}.gz ~/tools/hisat2-2.2.1/hisat2_extract_splice_sites.py ${GTF_FILE} > genome.ss ~/tools/hisat2-2.2.1/hisat2_extract_exons.py ${GTF_FILE} > genome.exon sbatch build_index.sh

#### build_index.sh

#!/bin/bash #PBS -l nodes=4:ppn=16,walltime=6:00:00,vmem=100gb #PBS -m abe #PBS -M yue.july.xing@gmail.com #PBS -N build_index #PBS -o build_index.o #PBS -e build_index.e cd /N/project/IUMP/dongq10152020/FVB_mice ~/tools/hisat2-2.2.1/hisat2-build -p 6 genome.fa \ --ss genome.ss --exon genome.exon GRCm39.106_tran

## Alignment

For TruSeq stranded sample prep kits, the library type is specified as 'fr-firststrand'. (https://rnabio.org/module-09-appendix/0009/12/01/StrandSettings/)

Use pre-built grcm38_tran:

#### align38.sh

#!/bin/bash #PBS -l nodes=1:ppn=16,walltime=16:00:00,vmem=100gb #PBS -m abe #PBS -M yue.july.xing@gmail.com #PBS -N align38 #PBS -o align38.o #PBS -e align38.e cd /N/project/IUMP/dongq10152020/FVB_mice mkdir bam38 mkdir counts38 sed -n '1,6p' file.list | while read name do ~/tools/hisat2-2.2.1/hisat2 -p 6 --dta --rna-strandness RF -x grcm38_tran/genome_tran -1 fq_trim/${name}_1P.fq.gz -2 fq_trim/${name}_2P.fq.gz -S bam38/${name}.p.sam samtools view -bS bam38/${name}.p.sam | samtools sort - -o bam38/${name}.p.bam rm bam38/${name}.p.sam ~/tools/hisat2-2.2.1/hisat2 -p 6 --dta --rna-strandness RF -x grcm38_tran/genome_tran -U fq_trim/${name}_1U.fq.gz,fq_trim/${name}_2U.fq.gz -S bam38/${name}.u.sam samtools view -bS bam38/${name}.u.sam | samtools sort - -o bam38/${name}.u.bam rm bam38/${name}.u.sam samtools merge bam38/${name}.mg.bam bam38/${name}.p.bam bam38/${name}.u.bam rm bam38/${name}.p.bam rm bam38/${name}.u.bam samtools sort -o bam38/${name}.bam bam38/${name}.mg.bam rm bam38/${name}.mg.bam mkdir counts38/${name} ~/tools/stringtie-2.2.1.Linux_x86_64/stringtie bam38/${name}.bam -e -B --rf -o counts38/${name}/${name}.counts.gtf -v -p 6 -G Mus_musculus.GRCm38.84.gtf done

#### align39.sh

#!/bin/bash #PBS -l nodes=1:ppn=16,walltime=6:00:00,vmem=100gb #PBS -m abe #PBS -M yue.july.xing@gmail.com #PBS -N align39 #PBS -o align39.o #PBS -e align39.e cd /N/project/IUMP/dongq10152020/FVB_mice mkdir bam39 mkdir counts39 sed -n '1,6p' file.list | while read name do ~/tools/hisat2-2.2.1/hisat2 -p 6 --dta --rna-strandness RF -x GRCm39.106_tran/GRCm39.106_tran -1 fq_trim/${name}_1P.fq.gz -2 fq_trim/${name}_2P.fq.gz -S bam39/${name}.p.sam samtools view -bS bam39/${name}.p.sam | samtools sort - -o bam39/${name}.p.bam rm bam39/${name}.p.sam ~/tools/hisat2-2.2.1/hisat2 -p 6 --dta --rna-strandness RF -x GRCm39.106_tran/GRCm39.106_tran -U fq_trim/${name}_1U.fq.gz,fq_trim/${name}_2U.fq.gz -S bam39/${name}.u.sam samtools view -bS bam39/${name}.u.sam | samtools sort - -o bam39/${name}.u.bam rm bam39/${name}.u.sam samtools merge bam39/${name}.mg.bam bam39/${name}.p.bam bam39/${name}.u.bam rm bam39/${name}.p.bam rm bam39/${name}.u.bam samtools sort -o bam39/${name}.bam bam39/${name}.mg.bam rm bam39/${name}.mg.bam mkdir counts39/${name} ~/tools/stringtie-2.2.1.Linux_x86_64/stringtie bam39/${name}.bam -e -B --rf -o counts39/${name}/${name}.counts.gtf -v -p 6 -G Mus_musculus.GRCm39.106.gtf done

## Put outputs together and convert to counts table

cd /N/project/IUMP/dongq10152020/FVB_mice mkdir gtf39 cp counts39/*/*.counts.gtf gtf39 awk '{print $1,"\tgtf39/"$1".counts.gtf"}' file.list > gtflist39.txt python ~/tools/prepDE.py3 -i gtflist39.txt -g mouse_gene_count_matrix_39.csv -t mouse_transcript_count_matrix_39.csv

mkdir gtf38 cp counts38/*/*.counts.gtf gtf38 awk '{print $1,"\tgtf38/"$1".counts.gtf"}' file.list > gtflist38.txt python ~/tools/prepDE.py3 -i gtflist38.txt -g mouse_gene_count_matrix_38.csv -t mouse_transcript_count_matrix_38.csv

## Output

Raw data stored at /scratch/yxing4/FVB_mice/raw_fastq. E:\FVB_N_mice /N/project/IUMP/dongq10152020/FVB_mice

**05/16/2022**

**DE for FVB_N mice RNA-seq data**

**This file performs pairwise DE comparisons for the data**

**Index used: GRCm39**

**Prepare input**

options(stringsAsFactors=FALSE) setwd("E:/FVB_N_mice") library(DESeq2) library(ggplot2) library(IHW) cts <- as.matrix(read.csv("mouse_gene_count_matrix_39.csv",row.names="gene_id")) trt=read.csv("trts.csv",row.names=1) all(rownames(trt) %in% colnames(cts)) all(rownames(trt) == colnames(cts)) cts <- cts[, rownames(trt)] all(rownames(trt) == colnames(cts)) trt$Treatment=as.factor(trt$Treatment)

**DE**

dds <- DESeqDataSetFromMatrix(countData = cts, colData = trt, design = ~ Treatment) keep <- rowSums(counts(dds)) >= 10 dds <- dds[keep,] dds$Treatment <- relevel(dds$Treatment, ref="NTG_1") dds <- DESeq(dds)

l1_l=c("R120G_1","R120G_3","R120G_6") l2_l=c("NTG_1","NTG_3","NTG_6") for (i in 1:3) { l1=l1_l[i] l2=l2_l[i] print(l1) print(l2) #res <- results(dds, alpha=0.05) res <- results(dds, contrast=c("Treatment",l1,l2),alpha=0.05) resOrdered <- res[order(res$padj),] print(resOrdered) print(summary(resOrdered, alpha=0.05)) write.csv(resOrdered,paste0("Mouse_genes_39.",l1,"_vs_",l2,".csv"),quote=FALSE) resIHW <- results(dds, filterFun=ihw, contrast=c("Treatment",l1,l2), alpha=0.05) resIHWOrdered <- resIHW[order(resIHW$padj),] print(resIHWOrdered) print(summary(resIHW, alpha=0.05)) write.csv(resIHWOrdered,paste0("Mouse_genes_IHW_39.",l1,"_vs_",l2,".csv"),quote=FALSE) }

**Get transformed counts**

vsd <- vst(dds, blind=FALSE) rld <- rlog(dds, blind=FALSE) write.csv(assay(vsd),paste0("Mouse_genes_39_vst.csv"),quote=FALSE) write.csv(assay(rld),paste0("Mouse_genes_39_rlog.csv"),quote=FALSE)

**PCA plot**

png(paste0("Mouse_genes_39_pca.png"),width=3000,height=3000,res=300) nudge <- position_nudge(y = 1) z=plotPCA(vsd, intgroup=c("Treatment")) + geom_text(aes(label = name), position = nudge, size=3) plot(z) dev.off()

**Index used: grcm38**

**Prepare input**

options(stringsAsFactors=FALSE) setwd("E:/FVB_N_mice") library(DESeq2) library(ggplot2) library(IHW) cts <- as.matrix(read.csv("mouse_gene_count_matrix_38.csv",row.names="gene_id")) trt=read.csv("trts.csv",row.names=1) all(rownames(trt) %in% colnames(cts)) all(rownames(trt) == colnames(cts)) cts <- cts[, rownames(trt)] all(rownames(trt) == colnames(cts)) trt$Treatment=as.factor(trt$Treatment)

**DE**

dds <- DESeqDataSetFromMatrix(countData = cts, colData = trt, design = ~ Treatment) keep <- rowSums(counts(dds)) >= 10 dds <- dds[keep,] dds$Treatment <- relevel(dds$Treatment, ref="NTG_1") dds <- DESeq(dds)

l1_l=c("R120G_1","R120G_3","R120G_6") l2_l=c("NTG_1","NTG_3","NTG_6") for (i in 1:3) { l1=l1_l[i] l2=l2_l[i] print(l1) print(l2) #res <- results(dds, alpha=0.05) res <- results(dds, contrast=c("Treatment",l1,l2),alpha=0.05) resOrdered <- res[order(res$padj),] print(resOrdered) print(summary(resOrdered, alpha=0.05)) write.csv(resOrdered,paste0("Mouse_genes_38.",l1,"_vs_",l2,".csv"),quote=FALSE) resIHW <- results(dds, filterFun=ihw, contrast=c("Treatment",l1,l2), alpha=0.05) resIHWOrdered <- resIHW[order(resIHW$padj),] print(resIHWOrdered) print(summary(resIHW, alpha=0.05)) write.csv(resIHWOrdered,paste0("Mouse_genes_IHW_38.",l1,"_vs_",l2,".csv"),quote=FALSE) }

**Get transformed counts**

vsd <- vst(dds, blind=FALSE) rld <- rlog(dds, blind=FALSE) write.csv(assay(vsd),paste0("Mouse_genes_38_vst.csv"),quote=FALSE) write.csv(assay(rld),paste0("Mouse_genes_38_rlog.csv"),quote=FALSE)

**PCA plot**

png(paste0("Mouse_genes_38_pca.png"),width=3000,height=3000,res=300) nudge <- position_nudge(y = 1) z=plotPCA(vsd, intgroup=c("Treatment")) + geom_text(aes(label = name), position = nudge, size=3) plot(z) dev.off()

**Output**

E:\FVB_N_mice

**05/17/2022**

**DE for FVB_N mice RNA-seq data - more comparisons**

**This file adds more pairwise DE comparisons for the data**

**Index used: GRCm39**

**Prepare input**

options(stringsAsFactors=FALSE) setwd("E:/FVB_N_mice") library(DESeq2) library(ggplot2) library(IHW) cts <- as.matrix(read.csv("mouse_gene_count_matrix_39.csv",row.names="gene_id")) trt=read.csv("trts.csv",row.names=1) all(rownames(trt) %in% colnames(cts)) all(rownames(trt) == colnames(cts)) cts <- cts[, rownames(trt)] all(rownames(trt) == colnames(cts)) trt$Treatment=as.factor(trt$Treatment)

**DE**

dds <- DESeqDataSetFromMatrix(countData = cts, colData = trt, design = ~ Treatment) keep <- rowSums(counts(dds)) >= 10 dds <- dds[keep,] dds$Treatment <- relevel(dds$Treatment, ref="NTG_1") dds <- DESeq(dds)

l2_l=c("R120G_1","R120G_3","R120G_1","NTG_1","NTG_3","NTG_1") l1_l=c("R120G_3","R120G_6","R120G_6","NTG_3","NTG_6","NTG_6") for (i in 1:length(l1_l)) { l1=l1_l[i] l2=l2_l[i] print(l1) print(l2) #res <- results(dds, alpha=0.05) res <- results(dds, contrast=c("Treatment",l1,l2),alpha=0.05) resOrdered <- res[order(res$padj),] print(resOrdered) print(summary(resOrdered, alpha=0.05)) write.csv(resOrdered,paste0("Mouse_genes_39.",l1,"_vs_",l2,".csv"),quote=FALSE) resIHW <- results(dds, filterFun=ihw, contrast=c("Treatment",l1,l2), alpha=0.05) resIHWOrdered <- resIHW[order(resIHW$padj),] print(resIHWOrdered) print(summary(resIHW, alpha=0.05)) write.csv(resIHWOrdered,paste0("Mouse_genes_IHW_39.",l1,"_vs_",l2,".csv"),quote=FALSE) }

**Index used: grcm38**

**Prepare input**

options(stringsAsFactors=FALSE) setwd("E:/FVB_N_mice") library(DESeq2) library(ggplot2) library(IHW) cts <- as.matrix(read.csv("mouse_gene_count_matrix_38.csv",row.names="gene_id")) trt=read.csv("trts.csv",row.names=1) all(rownames(trt) %in% colnames(cts)) all(rownames(trt) == colnames(cts)) cts <- cts[, rownames(trt)] all(rownames(trt) == colnames(cts)) trt$Treatment=as.factor(trt$Treatment)

**DE**

dds <- DESeqDataSetFromMatrix(countData = cts, colData = trt, design = ~ Treatment) keep <- rowSums(counts(dds)) >= 10 dds <- dds[keep,] dds$Treatment <- relevel(dds$Treatment, ref="NTG_1") dds <- DESeq(dds)

l2_l=c("R120G_1","R120G_3","R120G_1","NTG_1","NTG_3","NTG_1") l1_l=c("R120G_3","R120G_6","R120G_6","NTG_3","NTG_6","NTG_6") for (i in 1:length(l1_l)) { l1=l1_l[i] l2=l2_l[i] print(l1) print(l2) #res <- results(dds, alpha=0.05) res <- results(dds, contrast=c("Treatment",l1,l2),alpha=0.05) resOrdered <- res[order(res$padj),] print(resOrdered) print(summary(resOrdered, alpha=0.05)) write.csv(resOrdered,paste0("Mouse_genes_38.",l1,"_vs_",l2,".csv"),quote=FALSE) resIHW <- results(dds, filterFun=ihw, contrast=c("Treatment",l1,l2), alpha=0.05) resIHWOrdered <- resIHW[order(resIHW$padj),] print(resIHWOrdered) print(summary(resIHW, alpha=0.05)) write.csv(resIHWOrdered,paste0("Mouse_genes_IHW_38.",l1,"_vs_",l2,".csv"),quote=FALSE) }

**Output**

E:\FVB_N_mice

**05/23/2022**

**Annotate and merge DE results**

**This file merges results and annotates the genes in the results**

**Note**

DESeq2 has a section in their documentation that states why adjusted p-values will be set to NA. The reasons include genes with outlier counts, having low mean expression values, and having all 0 counts. If a row is filtered by automatic independent filtering, for having a low mean normalized count, then only the adjusted p value will be set to NA. https://www.biostars.org/p/458488/

**Download annotation file**

cd /N/project/IUMP/dongq10152020/FVB_mice wget http://ftp.ensembl.org/pub/release-106/mysql/mus_musculus_core_106_39/gene.txt.gz gunzip gene.txt.gz cd wget http://ftp.ensembl.org/pub/release-84/mysql/mus_musculus_core_84_38/gene.txt.gz gunzip gene.txt.gz mv gene.txt gene38.txt mv gene38.txt /N/project/IUMP/dongq10152020/FVB_mice

**Annotate results**

setwd("/N/project/IUMP/dongq10152020/FVB_mice/DE39") options(stringsAsFactors=FALSE) d0=read.delim("/N/project/IUMP/dongq10152020/FVB_mice/gene.txt",header=FALSE,row.names=1) d0=d0[,c("V2","V10","V13")] fls=list.files(".","Mouse_genes_39") for (fl in fls) { d1=read.csv(fl) d1$ID=gsub("\\|.*","",d1[,1]) d2=merge(d1,d0,all.x=TRUE,by.x="ID",by.y="V13") d2=d2[order(d2$padj),] colnames(d2)[2]="Name" colnames(d2)[c(9,10)]=c("Biotype","Description") fl=gsub(".csv",".annot.txt",fl) write.table(d2,fl,quote=FALSE,row.names=FALSE,sep="\t") } fls=list.files(".","Mouse_genes_IHW_39") for (fl in fls) { d1=read.csv(fl) d1$ID=gsub("\\|.*","",d1[,1]) d2=merge(d1,d0,all.x=TRUE,by.x="ID",by.y="V13") d2=d2[order(d2$padj),] colnames(d2)[2]="Name" colnames(d2)[c(10,11)]=c("Biotype","Description") fl=gsub(".csv",".annot.txt",fl) write.table(d2,fl,quote=FALSE,row.names=FALSE,sep="\t") }

setwd("/N/project/IUMP/dongq10152020/FVB_mice/DE38") options(stringsAsFactors=FALSE) d0=read.delim("/N/project/IUMP/dongq10152020/FVB_mice/gene38.txt",header=FALSE,row.names=1) d0=d0[,c("V2","V11","V14")] fls=list.files(".","Mouse_genes_38") for (fl in fls) { d1=read.csv(fl) d1$ID=gsub("\\|.*","",d1[,1]) d2=merge(d1,d0,all.x=TRUE,by.x="ID",by.y="V14") d2=d2[order(d2$padj),] colnames(d2)[2]="Name" colnames(d2)[c(9,10)]=c("Biotype","Description") fl=gsub(".csv",".annot.txt",fl) write.table(d2,fl,quote=FALSE,row.names=FALSE,sep="\t") } fls=list.files(".","Mouse_genes_IHW_38") for (fl in fls) { d1=read.csv(fl) d1$ID=gsub("\\|.*","",d1[,1]) d2=merge(d1,d0,all.x=TRUE,by.x="ID",by.y="V14") d2=d2[order(d2$padj),] colnames(d2)[2]="Name" colnames(d2)[c(10,11)]=c("Biotype","Description") fl=gsub(".csv",".annot.txt",fl) write.table(d2,fl,quote=FALSE,row.names=FALSE,sep="\t") }

**Merge all comparisons**

setwd("/N/project/IUMP/dongq10152020/FVB_mice/DE39") options(stringsAsFactors=FALSE) #fls=list.files(".","Mouse_genes_39.*annot.*") fls=list.files(".","Mouse_genes_IHW_39.*annot.*") fl=fls[1] fls=fls[-1] d1=read.delim(fl) d1=d1[,c("Name","Biotype","Description","log2FoldChange","padj")] fl=gsub(".*39.","",fl) fl=gsub(".annot.txt","",fl) colnames(d1)[(ncol(d1)-1):ncol(d1)]=paste0(fl,c("_LFC","_padj")) d0=d1 for (fl in fls) { d1=read.delim(fl) d1=d1[,c("Name","log2FoldChange","padj")] fl=gsub(".*39.","",fl) fl=gsub(".annot.txt","",fl) colnames(d1)[(ncol(d1)-1):ncol(d1)]=paste0(fl,c("_LFC","_padj")) d0=merge(d0,d1,all=TRUE,by="Name") } #write.table(d0,"Mouse_genes_39.all.annot.txt",quote=FALSE,row.names=FALSE,sep="\t") write.table(d0,"Mouse_genes_IHW_39.all.annot.txt",quote=FALSE,row.names=FALSE,sep="\t")

setwd("/N/project/IUMP/dongq10152020/FVB_mice/DE38") options(stringsAsFactors=FALSE) fls=list.files(".","Mouse_genes_38.*annot.*") #fls=list.files(".","Mouse_genes_IHW_38.*annot.*") fl=fls[1] fls=fls[-1] d1=read.delim(fl) d1=d1[,c("Name","Biotype","Description","log2FoldChange","padj")] fl=gsub(".*38.","",fl) fl=gsub(".annot.txt","",fl) colnames(d1)[(ncol(d1)-1):ncol(d1)]=paste0(fl,c("_LFC","_padj")) d0=d1 for (fl in fls) { d1=read.delim(fl) d1=d1[,c("Name","log2FoldChange","padj")] fl=gsub(".*38.","",fl) fl=gsub(".annot.txt","",fl) colnames(d1)[(ncol(d1)-1):ncol(d1)]=paste0(fl,c("_LFC","_padj")) d0=merge(d0,d1,all=TRUE,by="Name") } write.table(d0,"Mouse_genes_38.all.annot.txt",quote=FALSE,row.names=FALSE,sep="\t") #write.table(d0,"Mouse_genes_IHW_38.all.annot.txt",quote=FALSE,row.names=FALSE,sep="\t")

**Set 1 for adjp<0.05, |LFC|>1 (2 fold change)**

setwd("/N/project/IUMP/dongq10152020/FVB_mice/DE39") options(stringsAsFactors=FALSE) cps=c("NTG_3_vs_NTG_1", "NTG_6_vs_NTG_1", "NTG_6_vs_NTG_3", "R120G_1_vs_NTG_1", "R120G_3_vs_NTG_3", "R120G_3_vs_R120G_1", "R120G_6_vs_NTG_6", "R120G_6_vs_R120G_1", "R120G_6_vs_R120G_3") #d1=read.delim("Mouse_genes_39.all.annot.txt") d1=read.delim("Mouse_genes_IHW_39.all.annot.txt") d2=d1[,1:3] for (cp in cps) { ss=ifelse((abs(d1[,paste0(cp,"_LFC")])>1 & d1[,paste0(cp,"_padj")]<0.05),1,0) d2[,cp]=ss } #write.table(d2,"Mouse_genes_39.LFC1adjp0.05.annot.txt",quote=FALSE,row.names=FALSE,sep="\t") write.table(d2,"Mouse_genes_IHW_39.LFC1adjp0.05.annot.txt",quote=FALSE,row.names=FALSE,sep="\t")

setwd("/N/project/IUMP/dongq10152020/FVB_mice/DE38") options(stringsAsFactors=FALSE) cps=c("NTG_3_vs_NTG_1", "NTG_6_vs_NTG_1", "NTG_6_vs_NTG_3", "R120G_1_vs_NTG_1", "R120G_3_vs_NTG_3", "R120G_3_vs_R120G_1", "R120G_6_vs_NTG_6", "R120G_6_vs_R120G_1", "R120G_6_vs_R120G_3") #d1=read.delim("Mouse_genes_38.all.annot.txt") d1=read.delim("Mouse_genes_IHW_38.all.annot.txt") d2=d1[,1:3] for (cp in cps) { ss=ifelse((abs(d1[,paste0(cp,"_LFC")])>1 & d1[,paste0(cp,"_padj")]<0.05),1,0) d2[,cp]=ss } #write.table(d2,"Mouse_genes_38.LFC1adjp0.05.annot.txt",quote=FALSE,row.names=FALSE,sep="\t") write.table(d2,"Mouse_genes_IHW_38.LFC1adjp0.05.annot.txt",quote=FALSE,row.names=FALSE,sep="\t")

**Output**

E:\FVB_N_mice\annot E:\FVB_N_mice\merged /N/project/IUMP/dongq10152020/FVB_mice/DE38 /N/project/IUMP/dongq10152020/FVB_mice/DE39
