# Supplementary material for: Transcriptome remodeling of mouse hearts during postnatal cardiac maturation and under proteotoxic stress
Source: Mol Biol Rep. 2026 Feb 7;53(1):369. doi: 10.1007/s11033-026-11535-1 (PMC12882862; doi:10.1007/s11033-026-11535-1)
Supplement: Supplementary file 1 — Supplementary Material 1 [file 11033_2026_11535_MOESM1_ESM.zip › SBK2 RNA seq Supplementals/Figure S3. Western blot membranes and whole gels.docx]

**Figure S3. Western blot membranes and whole gels**

**
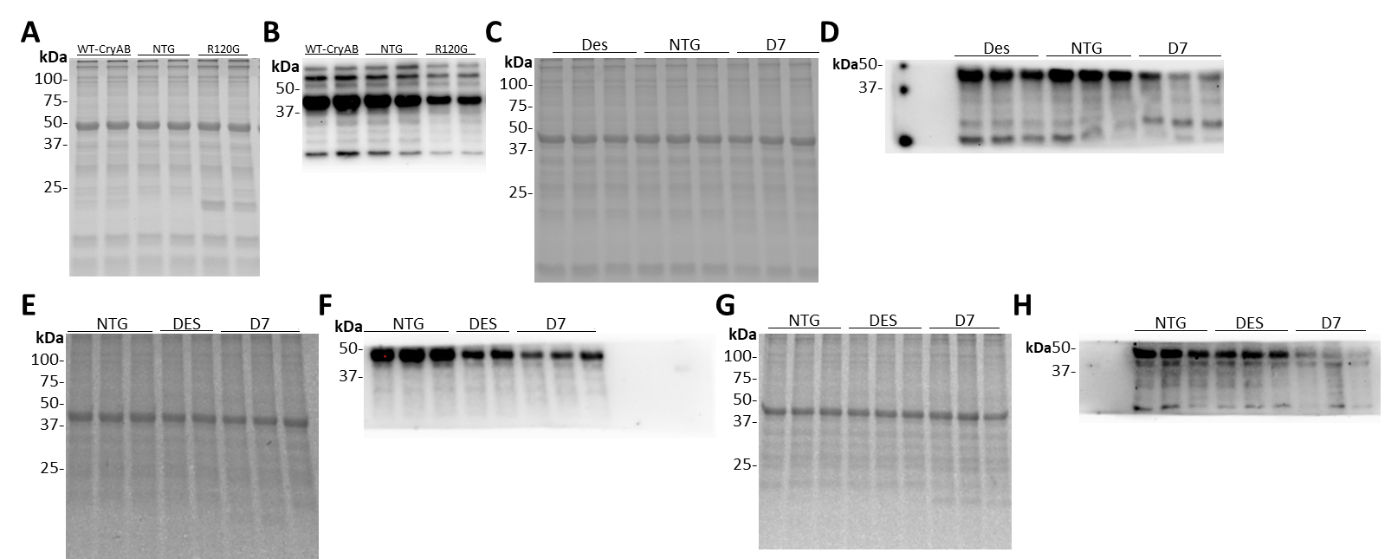
**

**Figure S3. Western blot membranes and whole gels from figure 3.**

**(A)** Membranes and **(B)** gel from 1 month NTG, WT-CryAB, and R120G hearts. **(C)** Membrane and **(D)** gel for 1 month NTG, Des, and D7 hearts. **(E)** Membrane and **(F)** gel for 3 month NTG, Des, and D7 hearts. **(G)** Membrane and **(H)** gel for 6 month NTG, Des, and D7 hearts.
